# Beyond the Pulmonary Veins: The Influence of Pulsed Field Ablation on the Superior Vena Cava

**DOI:** 10.1002/joa3.70249

**Published:** 2025-12-08

**Authors:** Eiji Yoshida, Yusuke Sakamoto, Hiroyuki Osanai, Hiroshi Asano

**Affiliations:** ^1^ Department of Cardiology Tosei General Hospital Seto‐city Aichi Japan

**Keywords:** anatomical proximity, atrial fibrillation, pulmonary vein isolation, pulsed field ablation, right superior pulmonary vein, superior vena cava

## Abstract

**Background:**

Pulsed field ablation (PFA) selectively ablates myocardial tissue while minimizing damage to surrounding structures. However, its effects on the superior vena cava (SVC) during right superior pulmonary vein (RSPV) isolation remain unclear.

**Objective:**

This study aimed to assess the impact of PFA on the SVC and determine whether anatomical proximity contributes to conduction delay or isolation.

**Methods:**

This prospective, single‐center observational study analyzed 15 patients with paroxysmal atrial fibrillation (AF) who underwent PFA at our institution between December 2024 and February 2025. A 12‐F multipolar PFA catheter (FARAWAVE) was used. The shortest RSPV‐SVC distance was measured using preprocedural contrast‐enhanced CT and intraprocedural electroanatomic mapping.

**Results:**

Among the 15 patients, 10 (67%) exhibited PFA‐induced conduction abnormalities in the SVC, including conduction delay or partial isolation. Partial SVC isolation was observed in seven patients (47%), with three (20%) developing SVC‐originating AF that required additional SVC ablation. The shortest RSPV‐SVC distance on CT was significantly shorter in the impact group (6.25 ± 1.7 mm) than in the nonimpact group (9.7 ± 2.0 mm; *p* = 0.04). Electroanatomic mapping showed no significant difference in the application‐SVC distance between groups (*p* = 0.12). ROC analysis identified 7.15 mm as the cutoff for predicting SVC involvement (AUC = 0.86; 95% CI, 0.63–1.00).

**Conclusion:**

PFA for RSPV isolation can cause SVC conduction abnormalities, with anatomical proximity as a key determinant. Preprocedural CT assessment may predict unintentional SVC involvement, which may alter arrhythmogenicity. Further studies are needed to assess long‐term clinical implications.

## Graphical abstract

Pulsed field ablation at the right superior pulmonary vein frequently causes conduction delay or partial isolation of the superior vena cava. Anatomical proximity between the two structures, as assessed by preprocedural CT, predicts the likelihood of SVC involvement.

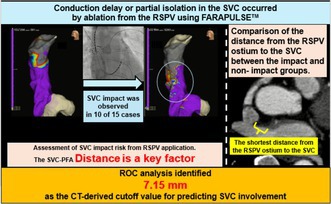

**Abbreviations**
AF: atrial fibrillation
LA: left atrium
PFA: pulsed field ablation
PVI: pulmonary vein isolation
RA: right atrium
RF: radiofrequency
RSPV: right superior pulmonary vein
SVC: superior vena cava

## 1 Introduction

Pulsed field ablation (PFA) creates an electric field around the catheter electrode by applying pulsed voltage, leading to selective myocardial ablation while sparing surrounding structures such as the phrenic nerve, esophagus, and pulmonary veins [1]. Myocardial cells are more susceptible to pulsed fields compared to the cells constituting these adjacent structures, allowing for selective myocardial ablation and reducing the risk of collateral damage [2]. In particular, recent reviews have emphasized that PFA combines myocardial tissue selectivity, a nonthermal mechanism, and tissue proximity dependency, and consistently demonstrates efficacy and safety comparable to conventional thermal ablation while reducing procedure time as well as the incidence of phrenic nerve and esophageal injury [3]. Previous studies have reported conduction delay in the superior vena cava (SVC) during right superior pulmonary vein (RSPV) isolation using radiofrequency (RF) ablation or cryoballoon ablation [4, 5]. This phenomenon is likely due to the anatomical proximity between the RSPV and the SVC, resulting in unintentional damage to the SVC during RSPV isolation. More recently, Matsuura et al. investigated the impact of PFA targeting the right pulmonary veins on the SVC and reported that conduction abnormalities may occur as an unintended consequence [6]. This finding suggests that, despite the favorable tissue selectivity of PFA, the close anatomical relationship between the RSPV and SVC may still result in inadvertent injury. In that study, conduction delay and electrical isolation in the SVC were observed during PFA for RSPV isolation, indicating potential SVC involvement.

In the present study, we aimed to clarify the extent to which PFA at the RSPV affects the SVC, to determine how anatomical proximity contributes to these effects, and to identify the most appropriate method for evaluating anatomical proximity as well as the cutoff distance at which such impact is likely to occur.

## 2 Methods

### 2.1 Study Design and Patients

This prospective, single‐center observational study evaluated patients who underwent ablation for paroxysmal AF in our hospital between December 2024 and February 2025. Patients contraindicated for contrast medium use due to renal dysfunction or allergy were excluded. A treatment plan was established for each patient based on preoperative contrast‐enhanced CT, evaluated by three independent electrophysiologists. Inclusion criteria were paroxysmal AF with simple anatomy that would optimize the FARAPULSE (Boston Scientific, USA) fit in the PV. The decision to perform PFA using the FARAPULSE system was made based on the size, number of branches, and anatomical shape of the pulmonary veins (PV), as assessed by preprocedural contrast‐enhanced CT. Patients with a common trunk or three branches in the pulmonary veins were excluded from the study. The study complies with the principles of the Helsinki Declaration (2013 revision) and was approved by the local institutional review board. Written informed consent was obtained from all participants prior to enrollment.

### 2.2 Ablation Protocol

Antiarrhythmic drugs were discontinued at least five half‐lives before the procedure. Ablation was performed under sedation with propofol and dexmedetomidine hydrochloride. Heparin was administered to maintain an activated clotting time (ACT) of ≥ 300 s during the procedure.

Surface electrocardiograms and bipolar intracardiac electrograms were continuously monitored and stored using a computer‐based digital recording system (LabSystem PRO, Boston Scientific, USA).

A 6F, 20‐pole dual‐site mapping catheter (BeeAT; Japan Lifeline Co Ltd., Tokyo, Japan) was inserted via the left subclavian vein and positioned in the coronary sinus, right atrium (RA), and SVC throughout the procedure. Additionally, an Optima 20‐pole ring catheter (St. Jude Medical, USA) was used to record LA and SVC potentials prior to pulmonary vein isolation (PVI).

A 12F multipolar PFA catheter (FARAWAVE; Boston Scientific, USA) was introduced into the left atrium (LA) via a steerable sheath (FARADRIVE; Boston Scientific, USA) and positioned at each PV ostium using a guidewire.

For each PV, eight pulse deliveries were applied, specifically: The catheter was configured in both “basket” and “flower” formations, delivering four applications per configuration, with a voltage setting of 2.0 kV. The catheter was rotated after every two pulse deliveries to ensure complete circumferential ablation.

To confirm entrance block, a five‐spline PFA catheter was inserted at each PV ostium, expanded into an “olive‐shaped” structure, and brought into contact with the surrounding tissue. This approach ensured complete PV isolation.

Three‐dimensional (3D) mapping was performed using an electroanatomic mapping system (EnSite Velocity; Abbott Laboratories, Abbott Park, IL, USA). Additional radiofrequency (RF) ablation was performed using a TactiFlex irrigated catheter (Abbott Laboratories, USA) as needed.

### 2.3 Electroanatomical Mapping and Anatomical Evaluation

SVC mapping was performed using an Optima 20‐pole ring catheter, and 3D mapping was conducted with the EnSite Velocity system (Abbott Laboratories, Abbott Park, IL, USA). Pre‐ and post‐ablation mappings were performed before and after PVI, respectively, to evaluate whether PFA at the RSPV induced conduction delay or isolation in the SVC. Simultaneous mapping during ablation was not performed, but both voltage and activation maps were created to visualize local conduction patterns. In addition, a 6F, 20‐pole BeeAT catheter was continuously positioned in the SVC throughout the procedure and served as a reference for identifying conduction delay or partial isolation. After completing all procedures, 3D mapping was also used to measure the shortest distance between the PFA application site and the SVC. Furthermore, contrast‐enhanced 320‐row CT was performed 5–10 days prior to the procedure to assess anatomical proximity. Specifically, the shortest distance from the RSPV ostium to the SVC was measured, and the SVC height was defined at the level of the left atrial roof on axial CT images. All image analyses were independently performed by three electrophysiologists (E.Y., Y.S., and H.O.), and the mean values were used to minimize measurement error.

### 2.4 Statistical Analysis

Continuous data were expressed as the mean ± standard deviation (SD) for the normally distributed variables and as the median (25th and 75th percentiles) for the non‐normally distributed variables. Welch's t‐test was used for comparisons, with statistical significance set at *p* < 0.05. Statistical analyses were performed using SPSS 22.0 for Windows (IBM Japan Ltd., Tokyo, Japan).

To evaluate the relationship between variables, the distance between the RSPV ostium and SVC was compared between patients with and without SVC impact (previously described as conduction delay or isolation). The optimal cutoff value for predicting SVC involvement was determined using receiver operating characteristic (ROC) curve analysis, selecting the threshold with the highest sensitivity and specificity. The area under the curve (AUC) was presented with its corresponding 95% confidence interval (CI).

## 3 Results

### 3.1 Baseline Patient Characteristics and Procedural Outcomes

A total of 15 patients (mean age, 70.0 ± 6.4 years; 53% male) were included. Patient characteristics are detailed in Table 1. All patients underwent successful EnSite mapping, and RSPV isolation was achieved exclusively with FARAPULSE.

**TABLE 1:** Patient characteristics (*n* = 15).

	Overall (*N* = 15)
Age (year)	70.0 ± 6.4
Male	8 (53%)
CHA_2_DS_2_ score	1.0 ± 1.3
CHA_2_DS_2_‐VASc score	2.0 ± 1.5
Arterial hypertension	7 (47%)
Diabetes mellitus	3 (20%)
Heart failure (HF)	1 (7%)
Stroke or TIA	1 (7%)
LVEF (%)	66.0 ± 8.1
LAD	36.0 ± 5.6
proBNP	109 ± 537.2
eGFR	63.0 ± 11.9

Abbreviations: LAD, left atrial diameter; LVEF, left ventricular ejection fraction; proBNP, pro brain natriuretic peptide.

### 3.2 Impact on the SVC by PFA

**FIGURE 1:**
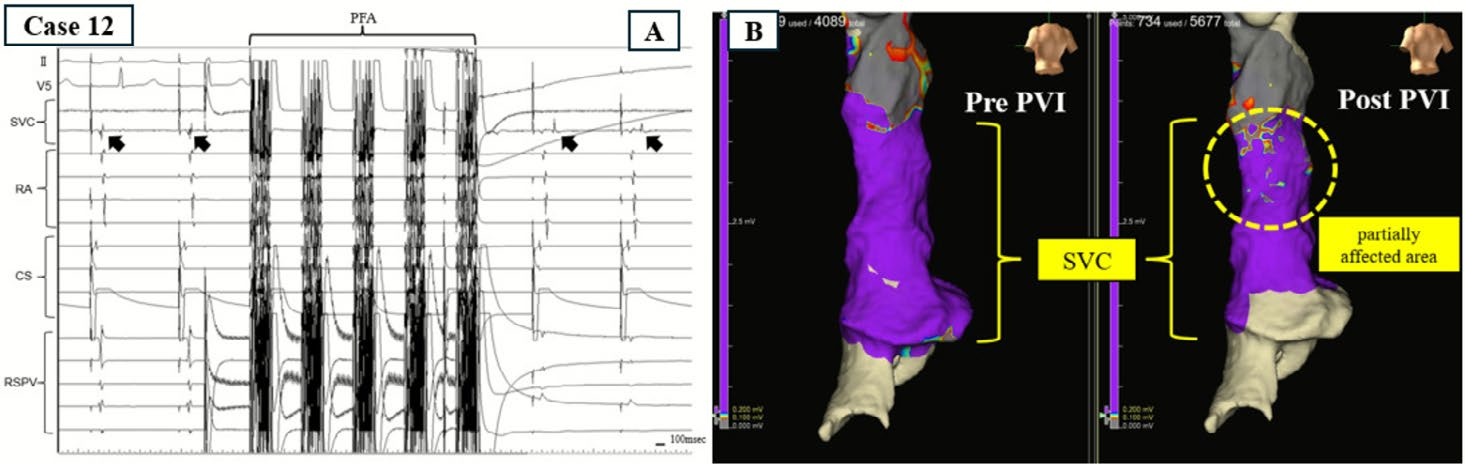
(A) In Case 12, intracardiac electrograms during PFA of the RSPV showing the loss of pulmonary vein potentials and delayed signals in the SVC (arrows) after the first application. CS, coronary sinus; PFA, pulsed field ablation; RA, right atrium; RSPV, right superior pulmonary vein; SVC, superior vena cava. (B) The left image shows the state before ablation of the RSPV, and the right image shows the state after ablation. A portion of the posterior wall of the SVC affected by PFA showed localized electrical alteration.

**FIGURE 2:**
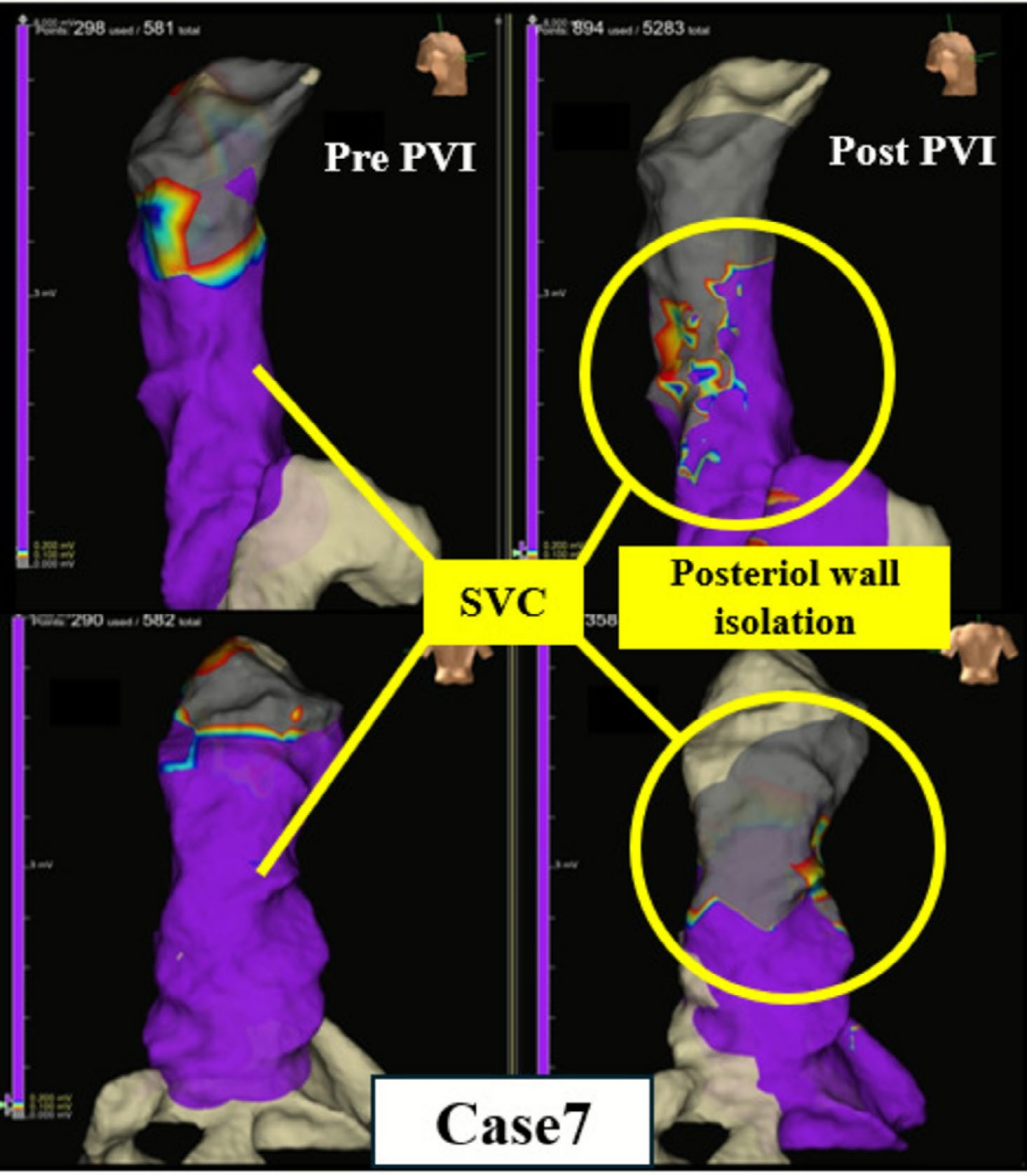
In Case 7, the posterior wall of the SVC was partially isolated. The left image shows the state before ablation of the RSPV, while the right image shows the state after ablation of the RSPV. The voltage disappeared in the posterior wall of the SVC, which corresponded to the anterior aspect of the RSPV.

Pre‐ and post‐ablation evaluations using the EnSite mapping system were performed to determine whether PFA applications at the RSPV had an impact on the SVC. Patients were divided into two groups based on the presence or absence of SVC impact. The shortest distance between the application site and the SVC was measured for each case and summarized in Table 2. Among patients in whom PFA impacted the SVC, the effect was classified as either conduction delay or partial isolation. Additionally, AF originating from premature atrial contractions (PACs) within the SVC was observed in three patients immediately after RSPV applications. The PACs that triggered AF showed the earliest activation on the catheter positioned in the SVC. These events were defined as “SVC‐triggered AF” and required additional RF ablation targeting the SVC. Additional SVC isolation was performed after completion of PV isolation. The location of the sinus node region was identified by determining the earliest activation site on the 3D activation map. After positioning of a 20‐pole circular catheter inside the SVC, an ablation line was created approximately 1 cm above the sinus node and below the circular catheter. The isolation line was constructed by delivering point‐by‐point energy in a counterclockwise direction from the anterior‐medial segment in the superior view until the SVC potentials were eliminated. RF ablation was also applied to regions that had become low voltage areas as a result of the impact of PFA. Finally, exit block from the SVC was confirmed in these patients. No additional RF ablation was performed in the remaining patients, as RF energy was used only for SVC ablation, even in the three patients who required it, and PVI was completed solely with PFA. In Case 12, intracardiac electrograms demonstrated conduction delay in the SVC immediately after PFA at the RSPV, although complete disappearance of the SVC potentials was not observed (Figure 1). In contrast, in Case 7, disappearance of the posterior SVC potentials was observed after ablation of the RSPV (Figure 2). These findings suggest that PFA at the RSPV had an impact on the SVC.

**TABLE 2:** The distance from the application site to the SVC was measured using the Ensite mapping system for each case, and the distance from the SVC to the RSPV ostium was measured using CT imaging.

Case	Age	Sex	Distance between the SVC to application on EnSite (mm)	Distance between the SVC and RSPV ostium on CT (mm)	SVC conduction abnormality
1	59	Male	1.0	9.8	Partial isolation of the posterior wall of the SVC AF originating from the SVC
2	66	Female	1.0	6.2	Partial isolation of the posterior wall of the SVC AF originating from the SVC
3	72	Female	4.0	6.6	Partial isolation of the posterior wall of the SVC AF originating from the SVC
4	68	Male	4.0	7.6	Partial isolation of the posterior wall of the SVC
5	66	Male	6.0	3.8	Partial isolation of the posterior wall of the SVC
6	65	Male	20.0	12.5	No impact
7	73	Female	4.0	3.9	Partial isolation of the posterior wall of the SVC
8	69	Male	16.0	9.8	No impact
9	78	Female	11.0	9.7	No impact
10	79	Female	7.0	7.2	No impact
11	80	Female	6.0	6.1	Partial isolation of the posterior wall of the SVC
12	74	Female	6.0	6.0	Conduction delay
13	61	Male	9.0	6.3	Conduction delay
14	76	Male	7.0	7.1	Conduction delay
15	70	Male	2.0	8.0	No impact

*Note:* Additionally, the damage on the SVC was summarized.

### 3.3 Electroanatomical Mapping and CT Analysis

**FIGURE 3:**
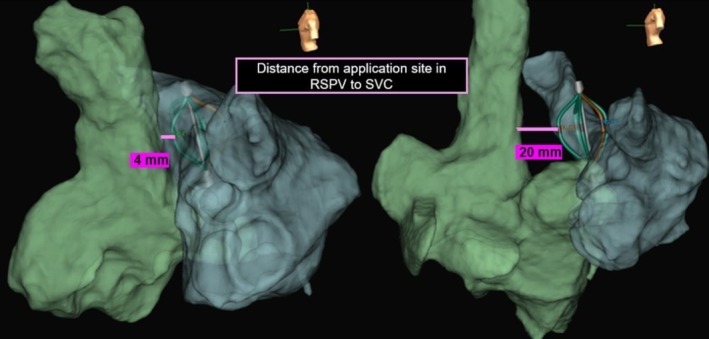
Using EnSite mapping, the shortest application‐SVC distance was measured in Case 6 and Case 7. Case 6: Application‐SVC shortest distance = 20.0 mm (right). Case 7: Application‐SVC shortest distance = 4.0 mm (left).

**FIGURE 4:**
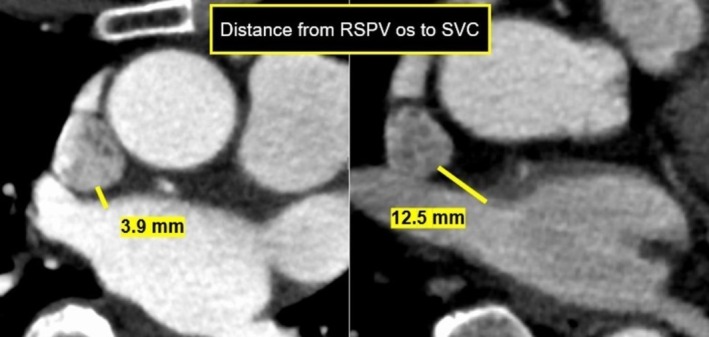
CT analysis revealed: Case 6: RSPV‐SVC shortest distance = 12.5 mm (right). Case 7: RSPV‐SVC shortest distance = 3.9 mm (left). The SVC height was defined as the level of the LA roof, and evaluated on axial CT images.

Using the EnSite mapping, the shortest application‐SVC distance was measured in Case 7 and Case 6, with Case 7 showing a distance of 4 mm and Case 6 showing a distance of 20 mm (Figure 3). Similarly, CT analysis (Figure 4) revealed that the shortest RSPV‐SVC distance was 3.9 mm in Case 7 and 12.5 mm in Case 6. Among the 15 patients, 10 (67%) exhibited PFA‐induced conduction changes in the SVC, including conduction delay or partial isolation. In all cases, isolation of the RSPV was confirmed by performing left atrial mapping prior to SVC mapping. SVC impact was defined as the occurrence of either conduction delay or partial isolation. Conduction delay was defined as a ≥ 10‐ms prolongation of the local electrogram recorded by the catheter positioned in the SVC after RSPV applications, without the formation of a new low‐voltage area on the post‐ablation map. Partial isolation was defined as the new appearance of a low‐voltage area ≥ 0.5 cm^2^ with bipolar electrograms < 0.5 mV during sinus rhythm after PVI using PFA. We confirmed the disappearance of potentials in the posterior wall of the SVC, indicating an entrance block. In cases where the voltage in the posterior SVC wall was extensively diminished, pacing at an output of 10 V from the low‐voltage area failed to capture the atrium, confirming an exit block. On the mapping catheter placed in the SVC, near‐field SVC potentials usually consisted of sharp components, and far‐field RA potentials consisted of dull components. Partial SVC isolation was observed in seven patients (47%), among whom 3 (20%) developed AF triggered by PACs originating from the SVC region during the initial phase of this study. Conduction delay was identified in three patients (20%), while the remaining five patients (33%) exhibited no conduction changes in the SVC.

### 3.4 Comparison Between the Impact and Nonimpact Groups

We compared the group in which PFA impacted the SVC, causing conduction delay or isolation (10 cases), with the group in which PFA had no impact on the SVC (five cases) (Table 3).

**TABLE 3:** In the Ensite‐measured application‐SVC distance, the impact group had a distance of 5.0 ± 2.5 mm, while the nonimpact group had 11.0 ± 7.1 mm (*p* = 0.12), showing no statistically significant difference.

	Impact group (*N* = 10)	Nonimpact group (*N* = 5)	*p*
Ensite‐measured Application‐SVC distance (mm)	5.0 ± 2.5	11.0 ± 7.1	0.12
CT‐measured SVC‐RSPV distance (mm)	6.25 ± 1.7	9.7 ± 2.0	0.04

*Note:* In the CT‐measured SVC‐RSPV ostium distance, the impact group had 6.25 ± 1.7 mm, whereas the nonimpact group had 9.7 ± 2.0 mm (*p* = 0.04), demonstrating a statistically significant difference.

**FIGURE 5:**
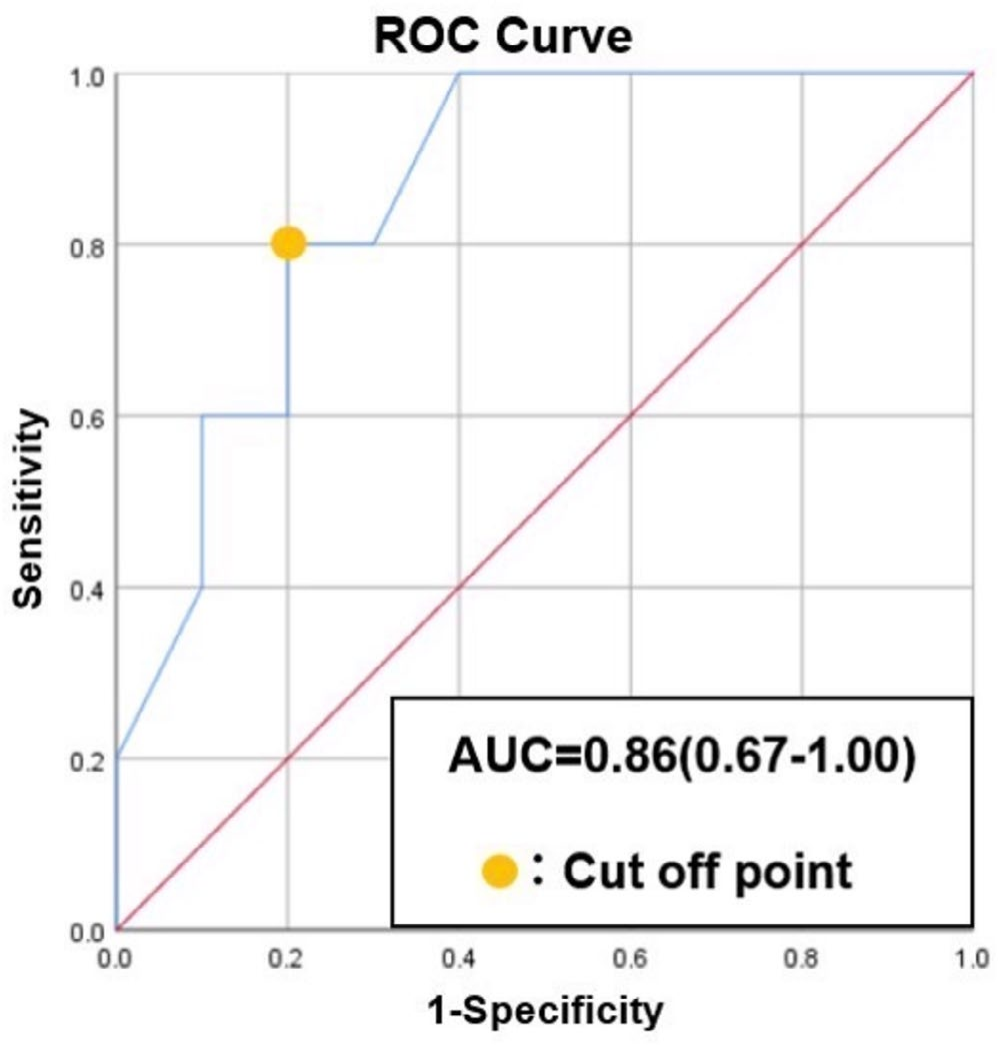
The effect of PFA at the RSPV on the SVC was evaluated by measuring the shortest distance on CT, with results analyzed using an ROC curve. The AUC was 0.86, with a 95% confidence interval (CI) of 0.63–1.00. The cutoff value was determined to be 7.15 mm, with a sensitivity of 80% and specificity of 80%.

The CT‐measured shortest distance between the RSPV and SVC was significantly shorter in the impact group (6.25 ± 1.7 mm) than in the nonimpact group (9.7 ± 2.0 mm, *p* = 0.04), indicating a strong association between anatomical proximity and SVC involvement. In contrast, the EnSite—measured Application‐SVC distance showed no statistically significant difference between the impact group (5.0 ± 2.5 mm) and the nonimpact group (11.0 ± 7.1 mm, *p* = 0.12), suggesting that electroanatomic mapping may be less accurate for predicting SVC involvement. ROC curve analysis (Figure 5) demonstrated that the shortest distance measured on CT was a strong predictor of SVC involvement following PFA at the RSPV. The AUC was 0.86 (95% CI: 0.63–1.00), indicating high predictive accuracy. The optimal cutoff value was determined to be 7.15 mm, with a sensitivity of 80% and specificity of 80%.

### 3.5 Clinical Outcomes

Among the 15 patients included in this study, no procedural complications occurred. During the 3‐month follow‐up period, AF recurrence was documented in one patient from the impact group and one patient from the nonimpact group; however, no redo procedures were performed. Importantly, in the three patients in whom SVC‐triggered AF was identified and treated with additional RF ablation targeting the SVC, no recurrence was observed during follow‐up.

## 4 Discussion

### 4.1 Main Findings

Although few studies have evaluated the impact of PFA on the SVC during RSPV ablation, several similar reports have been published. In particular, Matsuura et al. reported that PFA affected the SVC in 82.4% of cases (88.0% with the FARAPULSE system), which is consistent with the high incidence observed in our study [6]. Furthermore, both the present findings and previous reports suggest that the anatomical proximity between the RSPV and SVC correlates with the likelihood of SVC involvement. Specifically, in our study, the shortest distance between the RSPV ostium and the SVC measured on CT was identified as a cutoff value of 7.15 mm, indicating that PFA may impact the SVC when this distance is equal to or less than the threshold. In addition, CT‐based measurement proved to be an effective tool for predicting this distance, whereas prediction using the EnSite system was less reliable for assessing the risk of SVC involvement.

### 4.2 Collateral Effects of RSPV Ablation: SVC Conduction and Anatomical Proximity

Anatomically, the SVC and RSPV are highly adjacent, with the posterior aspect of the SVC being contiguous with the anterior aspect of the RSPV [7]. Previous studies have shown that SVC‐originating potentials can be recorded within the RSPV in up to 23% of patients, and vice versa, indicating potential cross talk between these structures [8].

Impact on SVC conduction caused by RSPV isolation has been reported with RF ablation [4], cryoballoon ablation [5], and HOT balloon ablation [9]. Miyazaki et al. examined the impact of second‐generation cryoballoon (CB) ablation on the SVC, suggesting that a shorter anatomical distance between the RSPV and SVC increases the likelihood of causing an impact on the SVC [4].

This study investigated the impact on the SVC caused by RSPV isolation using PFA. Compared to RF or cryoballoon ablation in previous reports, PFA may cause a greater impact on the SVC. PFA generates an electric field around the electrode by applying pulsed voltage, selectively affecting myocardial cells and inducing lesion formation [2]. Consequently, PFA tends to create larger lesions compared to conventional thermal ablation methods that directly apply heat energy.

Furthermore, previous studies have reported successful SVC isolation using PFA [10], suggesting that PFA could be effectively utilized for SVC ablation as well.

This study exclusively utilized the FARAPULSE system; however, previous studies have reported SVC isolation with PFA using the VARIPULSE system (Biosense Webster Inc., Irvine, CA) and the PulseSelect system (Medtronic, Minneapolis, MN), indicating that similar effects on the SVC may occur, although with varying frequencies [6].

Studies using a porcine heart model have demonstrated that greater electrode‐to‐tissue distance results in decreased lesion depth and width [11]. These findings suggest that anatomical proximity plays a crucial role in lesion formation, which may explain why PFA applied to the RSPV impacts the SVC.

### 4.3 Clinical Implications

PFA is highly effective for PVI; however, it may also cause an impact on the arrhythmogenic substrate of the SVC. The SVC is a well‐recognized source of non‐PV triggers due to its embryologic origin. Studies have shown that approximately 6% of patients with paroxysmal AF exhibit AF triggered by ectopic beats originating from the SVC [12].

Additionally, myocardial sleeve into the SVC has been demonstrated to have arrhythmogenic potential owing to its distribution and the heterogeneous arrangement of myocardial fibers [13]. Furthermore, PFA‐induced myocardial remodeling or conduction abnormalities in the SVC, which is already predisposed to serving as an AF trigger, may contribute to the formation of new AF triggers. Clinicians should be vigilant about the potential involvement of the SVC in cases of postprocedural AF recurrence.

Preprocedural CT imaging can be a useful tool for assessing the anatomical distance between the RSPV and SVC, potentially predicting the impact on the SVC. In this study, a cutoff value of 7.15 mm or less was identified as a threshold below which conduction delay or isolation may occur in the SVC due to PFA. Therefore, operators should be aware that PFA at the RSPV may have a potential impact on the SVC when this anatomical proximity exists.

On the other hand, despite these anatomical considerations, EnSite mapping did not reveal significant differences. This may be attributed to technical limitations, which may have influenced the results and led to the absence of significant differences. Notably, in Case 15, the EnSite display of FARAPULSE deviated significantly from the RSPV, highlighting the limitations of mapping technology that should be taken into account.

Additionally, we considered the short distance between the SVC and the RSPV ostium observed on CT as a potential predictive factor for the impact on the SVC. At our institution, during “basket”‐shaped applications, we intentionally perform applications near the RSPV ostium and avoid applying energy too deep inside the RSPV. Upon reviewing CT images, we observed cases where the SVC and RSPV ostium were well separated, whereas in some cases, the SVC was in contact with the deeper portion of the RSPV. This suggests that applications performed too deep within the RSPV may contribute to SVC impact. Nevertheless, some degree of SVC impact may be unavoidable in order to achieve complete isolation of the RSPV. The important point is to recognize that any impact on the SVC may alter its arrhythmogenic potential.

### 4.4 Study Limitations

This study has several limitations. First, the most significant limitation is the small sample size. Therefore, the interpretation of our findings should be approached with caution. Although a cutoff value of 7.15 mm was identified as a possible threshold below which SVC conduction delay or isolation may occur due to PFA, the wide confidence interval of the ROC analysis (AUC 0.63–1.00) indicates that this finding should be considered hypothesis‐generating and requires validation in larger cohorts.

Second, both EnSite mapping and CT‐based distance measurements were performed manually, potentially introducing some degree of measurement error.

Third, AF induction post‐PVI was not performed, which limited the ability to analyze AF triggers associated with SVC conduction delay or isolation. While the precise mechanism remains unclear, SVC‐triggered AF was observed consecutively in three initial cases. However, as more cases were accumulated, no further instances of SVC‐triggered AF were observed. Tohoku et al. reported roof‐dependent atrial tachycardia in patients with an arrhythmogenic posterior wall isthmus created by PFA [14], and Matsuura et al. proposed that SVC impact could be arrhythmogenic under certain conditions [6]. However, there are no existing data on the arrhythmogenicity of SVC impact. Further studies with larger sample sizes are necessary to clarify the electrophysiological consequences of PFA on the SVC. Future studies incorporating AF induction protocols could provide additional insights into the arrhythmogenic role of the SVC following PFA.

Finally, it remains uncertain whether the conduction delay and isolation observed in the SVC are transient or persist over the long term. This limitation stems from the design of the present study, which primarily focused on acute‐phase changes rather than long‐term outcomes. Consequently, it cannot be determined at this stage whether prophylactic intervention should be considered solely on the basis of SVC conduction delay, particularly in cases where the SVC has not been confirmed as the origin of AF. Previous case reports have suggested that SVC conduction delay or isolation may be transient [15]. Furthermore, as reported by Tanaka et al., the electrophysiological characteristics of the SVC are not uniform across patients, and some individuals may have preexisting conduction block lines within the SVC [16]. Although PFA may complete such preexisting block lines, the possibility that the observed findings reflect preexisting conduction abnormalities cannot be entirely excluded, and the extent to which PFA affects the SVC, as well as its underlying mechanisms, remains unclear. In light of these considerations, the potentially transient nature of these conduction changes should be carefully taken into account when interpreting their clinical significance. This remains an important clinical question, and further studies with extended follow‐up are warranted to determine the persistence and clinical implications of these findings.

## 5 Conclusion

PFA at the RSPV can cause conduction delay or partial isolation of the SVC, with anatomical proximity between the RSPV and SVC being a key determinant of these impacts.

Preprocedural CT imaging may help predict unintentional SVC impact, potentially contributing to arrhythmogenic remodeling and postprocedural AF recurrence. Further studies are needed to evaluate the long‐term effects of SVC impact induced by PFA.

## Author Contributions

Conceptualization: Eiji Yoshida, Yusuke Sakamoto, Hiroyuki Osanai. Data curation: Eiji Yoshida. Formal Analysis: Eiji Yoshida. Funding acquisition: None. Investigation: Eiji Yoshida. Methodology: Eiji Yoshida. Project administration: Eiji Yoshida, Yusuke Sakamoto. Resources: Eiji Yoshida. Software: Eiji Yoshida. Supervision: Yusuke Sakamoto, Hiroyuki Osanai. Validation: Eiji Yoshida. Visualization: Eiji Yoshida. Writing – original draft: Eiji Yoshida. Writing – review and editing: Eiji Yoshida. Eiji Yoshida: conception and design of the study, acquisition of data, analysis and interpretation of data, and manuscript drafting. Yusuke Sakamoto: conception and design of the study, technical help, writing, and editing assistance. Hiroyuki Osanai: conception and design of the study, technical help, writing, and editing assistance. Hiroshi Asano: technical help, writing, and editing assistance.

## Funding

The authors have nothing to report.

## Ethics Statement

Ethics approval All procedures performed in studies involving human participants were in accordance with the ethical standards of the institutional and/or national research committee and with the 1964 Helsinki Declaration and its later amendments or comparable ethical standards. The study was approved by the Bioethics Committee of the Tosei General Hospital (No. 1356).

## Consent

Informed consent was obtained from all individual participants included in the study.

## Conflicts of Interest

The authors declare no conflicts of interest.

## Supporting information


**Figure S1:** In Case 12, activation maps of the superior vena cava (SVC) before and after pulsed field ablation (PFA) targeting the right superior pulmonary vein (RSPV) are presented from two different views. Conduction delay is observed along the posterior wall of the SVC following ablation.

**Figure S2:** In Case 7, activation maps of the SVC before and after PFA of the RSPV are shown. The voltage disappeared in the posterior wall of the SVC, indicating partial isolation in the area corresponding to the anterior aspect of the RSPV.

## Data Availability

The authors have nothing to report.
