# Supplementary figures and images for: Beyond the Pulmonary Veins: The Influence of Pulsed Field Ablation on the Superior Vena Cava
Source: J Arrhythm. 2025 Dec 8;41(6):e70249. doi: 10.1002/joa3.70249 (PMC12685757; doi:10.1002/joa3.70249)

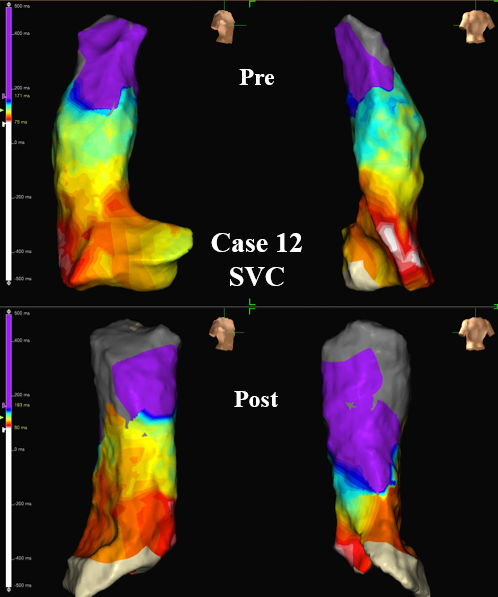

Supplement: Supplementary file 1 — Figure S1: In Case 12, activation maps of the superior vena cava (SVC) before and after pulsed field ablation (PFA) targeting the right superior pulmonary vein (RSPV) are presented from two different views. Conduction delay is observed along the posterior wall of the SVC following ablation. [file JOA3-41-e70249-s001.tif]

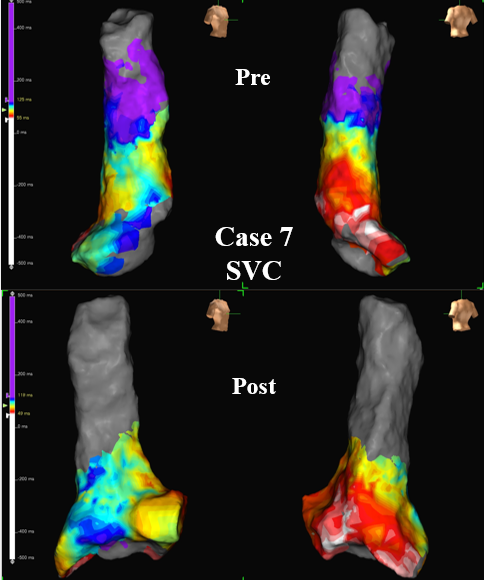

Supplement: Supplementary file 2 — Figure S2: In Case 7, activation maps of the SVC before and after PFA of the RSPV are shown. The voltage disappeared in the posterior wall of the SVC, indicating partial isolation in the area corresponding to the anterior aspect of the RSPV. [file JOA3-41-e70249-s002.tif]
